# Chronic social defeat alters brain vascular-associated cell gene expression patterns leading to vascular dysfunction and immune system activation

**DOI:** 10.1186/s12974-023-02827-5

**Published:** 2023-06-28

**Authors:** Joshua D. Samuels, Madison L. Lotstein, Michael L. Lehmann, Abdel G. Elkahloun, Subhadra Banerjee, Miles Herkenham

**Affiliations:** 1 Section on Functional Neuroanatomy, Intramural Research Program, National Institute of Mental Health, National Institutes of Health, Bethesda, MD 20892 USA; 2 Cancer Genetics and Comparative Genomics Branch, National Human Genome Research Institute, National Institutes of Health, Bethesda, MD 20892 USA; 3 Flow Cytometry Core, Laboratory of Genome Integrity, National Cancer Institute, National Institutes of Health, Bethesda, MD 20892 USA; 4 Neuroscience Graduate Program, Department of Neuroscience, Center for Brain Immunology and Glia, University of Virginia, 409 Lane Road, MR-4 6154, Charlottesville, VA 22908 USA

**Keywords:** Single-cell RNA sequencing, Cerebrovasculature, Psychosocial stress, Vascular repair, Immune activation

## Abstract

Brain vascular integrity is critical for brain health, and its disruption is implicated in many brain pathologies, including psychiatric disorders. Brain-vascular barriers are a complex cellular landscape composed of endothelial, glial, mural, and immune cells. Yet currently, little is known about these brain vascular-associated cells (BVACs) in health and disease. Previously, we demonstrated that 14 days of chronic social defeat (CSD), a mouse paradigm that produces anxiety and depressive-like behaviors, causes cerebrovascular damage in the form of scattered microbleeds. Here, we developed a technique to isolate barrier-related cells from the mouse brain and subjected the isolated cells to single-cell RNA sequencing. Using this isolation technique, we found an enrichment in BVAC populations, including distinct subsets of endothelial and microglial cells. In CSD compared to non-stress, home-cage control, differential gene expression patterns disclosed biological pathways involving vascular dysfunction, vascular healing, and immune system activation. Overall, our work demonstrates a unique technique to study BVAC populations from fresh brain tissue and suggests that neurovascular dysfunction is a key driver of psychosocial stress-induced brain pathology.

**Supplementary Information:**

The online version contains supplementary material available at 10.1186/s12974-023-02827-5.

## Introduction

The brain houses one of the most extensive vascular networks in the body. Uniquely, the cerebrovasculature is lined by a tightly regulated cellular structure, termed the blood–brain barrier (BBB), that prevents the direct interaction between the blood and the brain parenchyma [1]. In addition to the BBB, the blood-cerebrospinal fluid barrier separates blood from the cerebrospinal fluid (CSF) [1], and the arachnoid blood-cerebrospinal fluid barrier separates dural fenestrated blood vessels from the CSF in the subarachnoid space [2]. Brain-vascular barriers are a complex landscape with many different cell types contributing to their properties. For example, the well-studied BBB consists largely of brain endothelial cells—arterioles, capillaries, and venules which display unique tight junctions; vascular-associated mural cells—pericytes, smooth muscle cells, and fibroblasts; vascular-associated parenchymal cells—microglia, astrocytes, and neurons; and immune cells—resident perivascular macrophages [3–6]. Collectively, these brain vascular-associated cells (BVACs) constitute the neurovascular unit and have been shown to influence neuronal functioning within the brain parenchyma [7]. Dysfunction of brain-vascular barriers, most notably the BBB, is implicated in multiple neuropathologies, including stroke [8, 9], neurodegenerative diseases [10], aging [11, 12], traumatic brain injury [13, 14], and psychiatric disorders [15, 16]. Therefore, a better understanding of the BVAC landscape in health and disease is of high clinical and therapeutic importance.

To better understand the function of distinct cellular populations in brain homeostasis and pathology, single-cell RNA sequencing (scRNAseq) has emerged as a powerful way to assess the transcriptional profile of individual cells. Multiple recent studies have used scRNAseq to profile the mouse brain, including the neurovasculature, in homeostasis and disease states [17–19]. Yet, interrogating the subtle changes in BVACs in these studies has proven challenging due to difficulties surrounding their isolation and resulting low BVAC yields. Isolation methods like laser capture microdissection, magnetic bead isolation, and fluorescence-activated cell sorting have increasingly been used to isolate brain barriers [20–23], but these methods can be technically challenging and struggle to produce yields that represent the cellular complexity of brain barriers. The need for the development of techniques to isolate and enrich for BVACs is critical to understanding their complex roles in the brain.

Our interest in the brain-vascular interface arose from our studies indicating that chronic psychosocial stress in mice led to isolated incidences of BBB breakdown, manifested as microbleeds, and subsequent repair after stress cessation [24, 25]. These events appear to explain, in part, immune activation in the brain and periphery and correlate with increased depressive- and anxiety-like behaviors [26–28]. We hypothesized that cerebrovascular incidents, and resulting changes to BVAC populations, might occur also in human psychiatric diseases, notably those associated with psychosocial stress. Psychiatric disorders have complex etiologies. Their pathogenesis is driven by an intertwined set of biological, genetic, and environmental factors, and how these factors converge to contribute to pathology is still largely unknown. Affective disorders, like major depressive disorder (MDD), carry a large personal and societal disease burden, motivating the need for discoveries with the potential to inform therapeutic treatments. Recently, studies have identified brain-barrier dysfunction, most notably BBB disruption, as a player in MDD pathogenesis [29].

Psychosocial stress contributes to both MDD [30, 31] and vascular-related comorbid conditions such as hypertension and vascular disease [32, 33]. In animals, psychosocial stress can be studied using the chronic social defeat (CSD) paradigm, which carries validity [34] in modeling the effects of similar human stressors [35–38]. Mice subjected to CSD show depressive-like, anxiety-like, and asocial behaviors [36, 39]. While the cellular and mechanistic underpinnings for these changes are not well characterized, neuroinflammation, immune system involvement, and cerebrovascular dysfunction may be contributing factors. Notably, CSD causes an elevation of peripheral inflammatory cytokines [40–43], which may act on the brain vasculature and contribute to BBB breakdown and leakage of blood components like fibrin(ogen) into the brain [24, 43, 44]. We have proposed that leaked blood products may contribute to brain inflammation [24, 27] and microglial-mediated breakdown of the extracellular matrix and BBB [26] seen in CSD. These associations suggest that brain barrier disruption may be a critical driver of psychosocial stress-induced pathology. Yet, it is largely unclear how psychosocial stress impacts the complex cellular milieu of brain barriers.

To this end, we developed a novel method to isolate and enrich for BVACs from fresh mouse brains and compared BVAC transcriptional profiles between CSD and non-stress, home-cage (HC) conditions. Using anti-CD31 tagged magnetic microbeads followed by mechanical and enzymatic dissociation steps, we recovered multiple vascular, mural, and immune cell populations. scRNAseq confirmed the identities of these cell populations, including multiple transcriptionally distinct endothelial cell and microglia populations. Although BVAC ratios were unchanged between the HC and CSD conditions, CSD induced transcriptional changes in BVAC populations related to brain-barrier breakdown, vascular repair, and immune system activation. Thus, neurovascular dysfunction and immune signaling may play key roles in psychosocial stress-induced neuropathology.

## Methods

### Animals

All procedures were approved by the National Institute of Mental Health Institutional Animal Care and Use Committee and conducted in accordance with the National Institute of Health guidelines. Experimental procedures were performed using 12–14-week-old male C57BL/6 and male CD-1 retired breeders (Charles River Laboratories). All animals were pair-housed in pathogen-free conditions in a 12-h reversed light/dark cycle (lights off at 9:00 AM). Food and water were provided ad libitum.

### Chronic social defeat (CSD)

CSD was used to model the effects of chronic psychosocial stress in mice. C57BL/6 mice were randomly assigned to CSD (n = 8) or home cage (HC, n = 8) conditions. As conducted previously [25, 45], an experimental C57BL/6 mouse was housed for 14 days in the home cage of a novel, aggressive CD-1 mouse. The mice were separated by a perforated, transparent acrylic partition allowing for continuous olfactory, visual, and auditory sensory exchange between the animals. The partition was removed for 5 min each day to allow for agonistic encounters between the mice. Daily defeat encounters were closely monitored to ensure that reliable defeats occurred [39]. To minimize wounding during the encounters, the mandibular incisors of the CD-1 mouse were trimmed. If observable defeats did not occur, the experimental C57BL/6 mouse was transferred to the home cage of a novel aggressive CD-1 mouse, and agonistic encounters were allowed to occur for 5 min. Experimental animals in the HC condition were pair-housed for 14 days with a CD-1 mouse permanently separated from it by an acrylic partition.

### Social interaction (SI) test and behavioral analysis

Testing was conducted as previously described [24, 26]. Briefly, experimental C57BL/6 mice were placed in a 50 × 50 cm open field arena containing two perforated acrylic cylinders (10 cm diameter) centered in opposite quadrants. An acclimation trial was conducted prior to the testing trial in which both cylinders were empty. For the testing trial, a novel CD-1 mouse was placed inside one of the cylinders while the other remained empty. In both trials, experimental mice were placed in the center of the open field and allowed to explore for 5 min. Time spent investigating each cylinder was recorded from above and analyzed using TopScan (CleverSys Inc., Reston, VA). The SI quotient was calculated by dividing the time spent investigating the cylinder containing the CD-1 mouse by the time spent investigating the empty cylinder. Lower scores indicated asocial behavior.

**Fig. 1:**
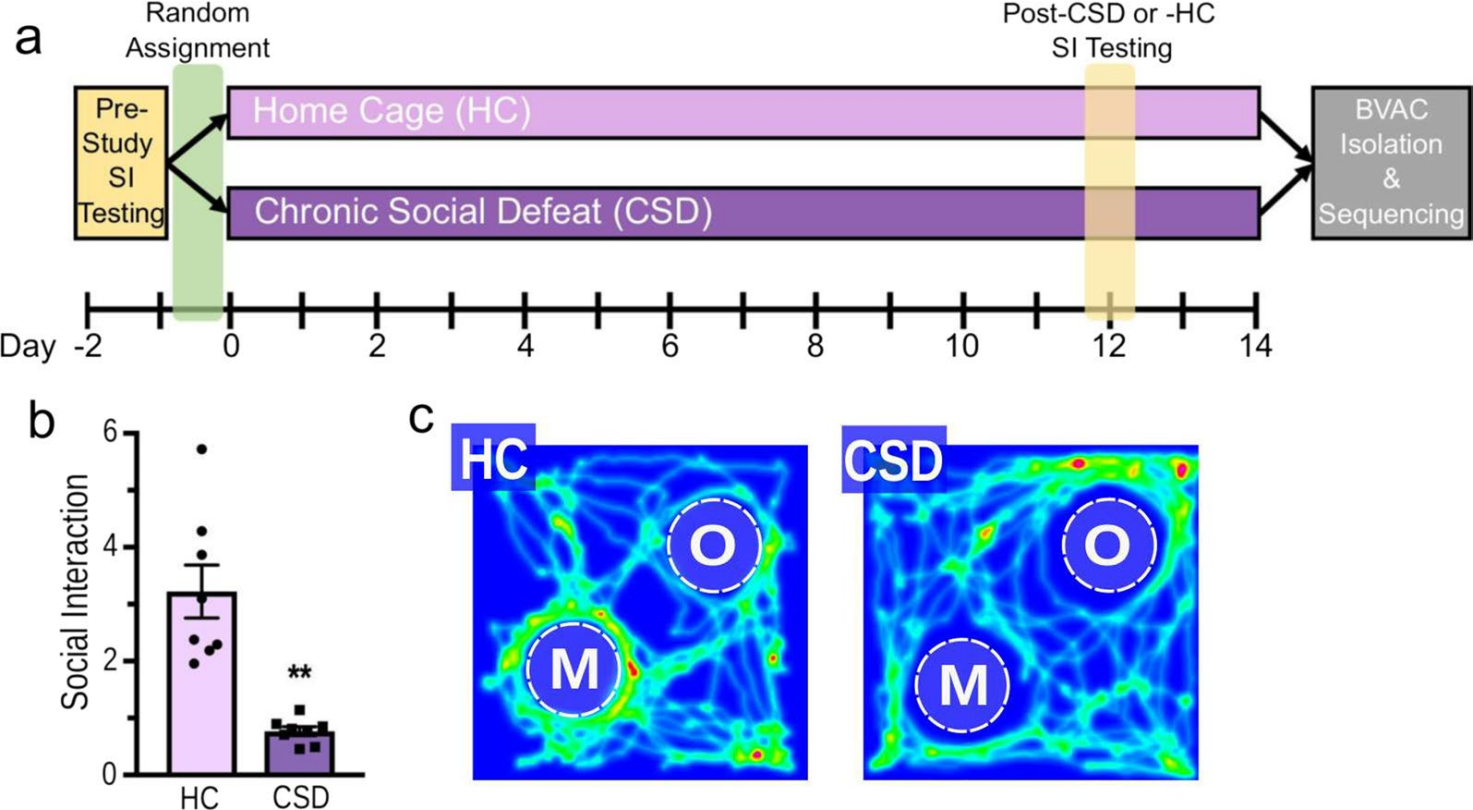
Chronic social defeat (CSD) results in reduced sociability. **a** The experimental timeline. **b** CSD-exposed mice showed significant reductions in social exploration in the social interaction (SI) test. The SI quotient was calculated by dividing the time spent investigating the cylinder containing the CD-1 mouse by the time spent investigating the empty cylinder. **c** Representative heat maps of social interaction behavior for CSD and HC mice in the SI task. *N* 8 per group, **p 0.001, *Error* *bars* standard error of the mean, *CSD* chronic social defeat, *HC* home cage, *SI* social interaction, *M* mouse, *O* object

C57BL/6 mice (n = 22) were phenotyped prior to the start of the study (Fig. 1a). Of the 22 mice, five mice were removed from the study due to low SI scores and one mouse was removed due to excessive time spent not interacting with the target cylinders (Additional file 1 Fig. S1). The remaining mice (n = 16) were randomly assigned into the experimental housing conditions (HC; n = 8, CSD; n = 8). All mice were phenotyped again 2 days prior to tissue harvest (CSD or HC day 12). Efficient CSD was confirmed in all 8 CSD mice as measured by reduced SI quotients compared to HC mice (Unpaired T-test with Welch’s correction, t = 5.22, p = 0.001, Fig. 1b). Heatmap tracing confirmed reduced social interaction in the CSD mice (Fig. 1c). For the CSD group, phenotyping occurred 20 h after the defeat exposure on the previous day. All phenotyping was conducted in the dark phase of the light cycle under dim red lighting (approximately 40 lx). Animals were acclimated to the behavioral testing room for at least 60 min prior to testing.

### Brain vascular-associated cell (BVAC) isolation

One day prior to tissue collection and neurovascular cell isolation, Dynabeads (referred to “beads” onward; Invitrogen, 11203D) were resuspended by tilting and rotating the vial for 5 min and 50 µl/brain were transferred to a clean Eppendorf tube containing 1 ml of 1X phosphate buffered saline (PBS) with 1% bovine serum albumin (BSA; Jackson ImmunoResearch, 001-000-162) and 2 mM etheylenediaminetetraacetic acid (EDTA; 0.5 M pH 8.0, Invitrogen AM9260G; full solution referred to as “washing medium” onward). The beads were washed by placing the tube on the DynaMag (Invitrogen DYNAL Bead Separator; referred to as “magnet”) for 1 min and discarding the supernatant. The tube was removed from the magnet and the beads were resuspended in the volume of washing medium equivalent to the beads (50 µl/brain). Ten microliters of CD31 antibodies (0.5 mg/µl; BD Biosciences, 553370) per brain were added to the tube. CD31 is a adhesion marker specifically found on endothelial cells within the brain and has been historically used to isolate brain endothelial cells [46]. The beads and antibodies were allowed to incubate overnight at 4 °C with gentle inversion using a carousel rotating shaker to allow for conjugation. While the overnight conjugation method was conducted for this study, the bead and antibody conjugation steps can also be performed as above on the day of tissue harvest and neurovascular isolation with a 1 h incubation and conjugation at room temperature.

On the day of brain harvest prior to brain collection, the tube containing the conjugated beads and CD31 antibodies was placed on the magnet for 1 min, and the supernatant was discarded. The beads were washed by removing the tube from the magnet, adding 1 ml of washing medium, placing the tube back on the magnet, and discarding the supernatant. The final conjugated beads were resuspended in 50 µl of washing medium per brain, maintained at room temperature, and used later in the isolation procedure. Additionally, collagenase II (Worthington, LS004176) was thawed to room temperature and an aliquot of Dulbecco’s Modified Eagle Medium (DMEM; 3 ml per brain; Gibco, 10,569–010) was brought to 37 °C in an incubator.

Stressed and non-stressed C57BL/6 mice were anesthetized with isoflurane and rapidly transcardially perfused with 24 ml of ice cold 1X PBS. Brains were removed and transferred into individual pre-chilled 15 mL conical tubes containing 10 ml of 1X PBS at 4 °C. Each brain was transferred to a sterile petri dish where the cerebellum, striatum, brainstem, olfactory bulbs, and extraneous white matter were removed using a sterile blade. The remainder of each brain was chopped with a sterile blade until no large pieces were visible. The chopped brains were transferred to individual 5 ml polystyrene round-bottom tubes using 3 ml of 37 °C DMEM with a cut p1000 pipette tip. No RNAse inhibitors were used during the isolation procedure. Twenty microliters of collagenase II (1 mg/ml) were added to each tube, mixed until the suspension passed smoothly through the tip of a cut p1000 pipette tip, and incubated for 10 min at 37 °C with gentle rotation. Each suspension was transferred to a new 15 ml conical tube using an additional 12 ml of room temperature DMEM. The samples were centrifuged at room temperature for 5 min at 300*g* and the liquid was aspirated. The remaining pellet was resuspended in 3 ml of room temperature DMEM and divided equally in three clean 1.5 ml Eppendorf tubes. The final conjugated beads (see above) were mixed via gentle pipette trituration and 15 µl of the conjugated beads were added to each sample-containing Eppendorf tube. The tubes containing the suspension and conjugated beads were allowed to incubate for 30 min at room temperature with gentle inversion using a carousel rotating shaker.

Following incubation, the tubes were placed on the magnet for 2 min, and the supernatant was removed and collected. The beads were washed 2 times by removing the tubes from the magnet, resuspending the beads in 1 ml of room temperature DMEM, triturating the suspension 5 times with a pipette, and placing back on the magnet for 2 min, each time removing and collecting the supernatant. This supernatant was pooled by condition and further processed in tandem with the bead-containing neurovascular samples as a negative control containing non-neurovascular cells. Following the final wash, the pellets from each triplicate of tubes per brain were pooled by resuspending the first pellet in 1 ml of room temperature DMEM and using the resuspension to resuspend and transfer the second and third pellets to a new Eppendorf tube. An additional 0.5 ml of DMEM was used to wash each triplicate of tubes to ensure a complete transfer. The pooled tubes were placed on the magnet for 2 min, the supernatant was removed, and the beads were resuspended in 1 ml of room temperature DMEM. Twenty microliters of collagenase II (3 mg/ml) were added to each tube, mixed by passing the suspension 10 times through a sterile 20 g needle attached to a sterile 1 ml syringe without creating bubbles, and incubated for 10 min at 37 °C with gentle rotation. The tubes were placed on the magnet for 2.5 min, and the supernatant was removed and collected in a new 15 ml conical tube. The beads were washed 3 times by removing the tubes from the magnet, resuspending the beads in 1 ml of room temperature DMEM, and placing the tubes back on the magnet for 2.5 min. For each wash, the supernatant was removed and collected in the corresponding 15 ml tube.

Each tube was filled to 5 ml with room temperature DMEM and subject to centrifugation at 4 °C for 5 min at 300 g. Samples were kept on ice or at 4 °C for the remaining steps. The liquid was aspirated, and each pellet was resuspended in 1080 µl of Hank’s buffered salt solution (HBSS; Gibco, 14025092) with 0.1% BSA and 2 mM EDTA (referred to as “HBSS/BSA/EDTA” onward). One-hundred twenty microliters of Myelin Removal Beads (Miltenyi Biotec, 130-096-733) were added to each tube and incubated for 15 min at 4 °C with no agitation. Each tube was filled to 5 ml with HBSS/BSA/EDTA, subject to centrifugation at 4 °C for 5 min at 300*g*, and resuspended in 1 ml of HBSS/BSA/EDTA. Two LS columns (Miltenyi Biotec) per sample were prepared by rinsing each with 3 ml of HBSS/BSA/EDTA on a magnet. Half of the pellet resuspension was passed through each column pair and the eluent was collected in new 15 ml conical tubes. The columns were washed 2 times with 1 ml of HBSS/BSA/EDTA, collecting the eluent each time. The eluents were combined into a single tube, subject to centrifugation at 4 °C for 5 min at 300*g*, and resuspended in 500 µl of HBSS with 0.1% BSA. Each sample was passed through and collected in a 5 ml polystyrene round-bottom tube with a 35 µm cell strainer cap (Falcon, 352,235). Each sample was further filtered through a 20 µm filter (Miltenyi Biotec, 130-101-812) and collected in a new 5 ml polystyrene round-bottom tube. The final filtered samples enriched for neurovascular-associated cells from 4 samples of the same condition and balanced SI scores were pooled and submitted for sequencing as an individual sample. Two replicate samples for each condition were submitted for sequencing.

### Single-cell RNA sequencing (scRNAseq)

Cell number and viability were checked with a Luna-FX7 (Logos Biosystems) cell counter. For scRNA-seq, gel bead-in-emulsions were prepared by loading up to 10,000 cells per sample onto the Chromium Chip G (10 × Genomics 1000073) and run using the Chromium Controller (10 × Genomics). cDNA libraries were generated with Chromium Single Cell 3′ GEM, Library and Gel Bead Kit V3.1 (10 × Genomics). Libraries were sequenced using the NextSeq 500/550 High Output Kit v2.5 (Illumina) on an Illumina NextSeq 550 sequencer. CellRanger version 6.0 (10 × Genomics) software was used for demultiplexing and generating the h5 files that were subsequently used by downstream analysis.

### Bioinformatic analysis

Data processing occurred using Seurat (version 4.0.2) and a custom R pipeline. Briefly, quality control and filtering were performed to remove cells containing a low number of reads (< 500 UMI) and genes (< 250 genes) and > 25% mitochondrial reads. Additionally, cells containing a low complexity score (< 0.8 log_10_GenesPerUMI) were removed. While doublets are often recovered in single-cell studies of vascular cells, we did not detect cells with abnormally high counts or genes, a common indicator of possible doublet contamination [47]. This suggests that our isolation method performed exceptionally well at dissociating closely connected neurovascular cells into a single cell suspension. After quality control and filtering steps, 12,744 cells remained. Subsequently, integration, normalization, and scaling steps were performed using SCTransform in Seurat to correct for batch effects. Principal component analysis was performed, and uniform manifold approximation and projection (UMAP) reduction was conducted using the top 40 principal components (PCs) based on the ElbowPlot. Cells were clustered using the FindNeighbors and FindClusters functions in Seurat, which use the Louvain clustering algorithm, and a resolution of 1.6 was chosen for downstream analysis. The FindAllMarkers function in Seurat was used to identify the differentially expressed genes corresponding to each cluster against all other clusters. Cells were arranged in 26 distinct clusters including multiple BVAC populations. One small cluster (329 cells) was identified as a doublet cell cluster based on shared gene expression pattern and was not included in downstream differential gene testing and pathway analysis. Endothelial and microglia clusters were further isolated, and clusters were re-analyzed using the FindAllMarkers function as previously described.

Gene differential expression analysis was performed using the FindMarkers function in Seurat and the wilcox algorithm which uses the Wilcoxon Rank Sum test to determine gene expression differences between CSD and HC. Resulting differentially expressed genes for each cluster were filtered to remove all genes with adjusted *P* values > 0.05 and were used for downstream pathway analysis. The Reactome pathway database (https://reactome.org/) and Gene Ontology: Biological Processes (PANTHER16.0, http://www.pantherdb.org/) were used to examine pathway enrichment associated with the differentially expressed genes corresponding to each cell cluster. For Reactome analyses, human projection was used, and possible interactors were not included. All data visualization was performed using Seurat and ggplot2 (version 3.3.3).

Cell–cell communication and ligand receptor interactions were probed using the CellChat package [48]. The Seurat object was converted to a CellChat object and the mouse CellChat database was used for predicting interactions. Significant (*P* < 0.05) cell–cell communication and ligand-receptor interactions were inferred using a permutation test and the recommended trimean method, respectively. Centrality scores were calculated by computing multiple network centrality measures including out-degree, in-degree, flow betweenness, and information centrality to identify communication senders, receivers, mediators, and influencers. Interrogation and visualization of the interaction network was performed using chord diagrams, heatmaps, ligand-receptor pair graphs, and bubble plots all within the CellChat package.

### Statistics

Behavioral data were summarized as the mean ± SEM. Differences between treatment conditions were considered statistically significant when the p value was < 0.05. An unpaired t-test using Welch’s correction accounted for different standard deviations between treatment conditions and was analyzed with GraphPad Prism 7.

## Results

### CD31 antibody-tagged magnetic microbead isolation preferentially enriches for BVACs

**Fig. 2:**
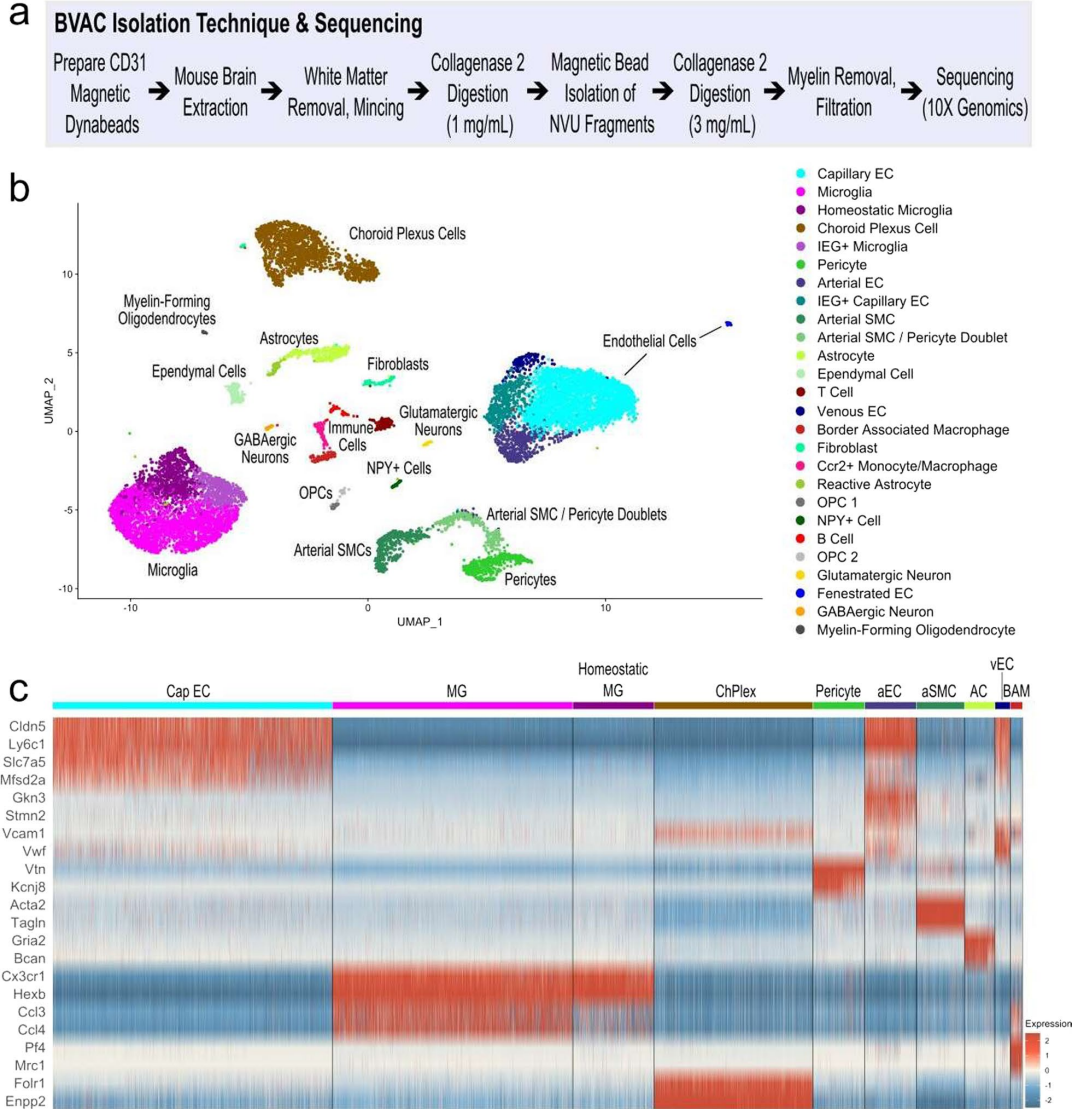
Magnetic bead isolation in mice in HC and CSD conditions enriches for BVACs. **a** Key steps in the neurovascular cell isolation method. **b** UMAP of 12,744 cells following QC and integration from all CSD and HC samples, color-coded based on cell identification. **c** Heatmap of signature gene expression compared to all other clusters for neurovascular unit cell types that comprise the key brain barriers (i.e., BBB). Increased expression is colored red, and decreased expression is colored blue. *QC* quality control; *UMAP* uniform manifold approximation and projection; *BBB*  blood brain barrier; *EC* endothelial cell; *IEG* immediate-early gene; *SMC* smooth muscle cell; *OPC* oligodendrocyte precursor cell; *CapEC* capillary endothelial cell; *MG* microglia; *ChPlex*  choroid plexus; *aEC* arterial endothelial cell; *AC* astrocyte; *vEC* venous endothelial cell; *BAM* border-associated macrophage

Due to poor isolation efficiency and low cellular yields, BVACs from fresh tissue can be difficult to characterize at the single-cell level. To address this challenge, we developed a BVAC enrichment protocol (Fig. 2a) to isolate and enrich for BVACs from fresh mouse brain tissue by anti-CD31 antibody-tagged magnetic-microbead isolation and mechanical and enzymatic digestion for droplet-based scRNAseq. We applied the protocol to 16 freshly extracted mouse brains from home cage (HC, n = 8) and chronic social defeat (CSD, n = 8) conditions. We pooled samples of the same condition into two groups of four for the final scRNAseq to increase BVAC yields per run. Following quality control and integration steps (see Methods, Bioinformatic Analysis), we obtained 12,744 cells. Similar cell quantities were captured from the pooled HC (6,749 cells) and CSD (5,995 cells) samples (Additional file 1: Fig. S2a), as evidenced by the overlap of the HC and CSD UMAPs. Similarly, cell quantities captured from individual HC and CSD runs showed similar overlap (Additional file 1: Fig. S2b).

Unsupervised clustering using Seurat and a custom R pipeline identified 26 distinct clusters, and a clear enrichment for endothelial and perivascular clusters (Fig. 2b). Signature gene profiles for individual cell clusters were used to identify each of the clusters (Additional file 1: Fig. S3, S4, Additional file 2), along with the lack of expression of signature genes corresponding to other clusters. All endothelial cell (EC) clusters showed high expression of canonical endothelial genes (i.e., Cldn5, Itm2a, Ly6c1, Ly6a, and Flt1) compared to other non-endothelial clusters. Similarly, we also identified multiple microglia populations all expressing signature microglial genes (i.e., Cx3cr1, Hexb, Tmem119, P2ry12, Csf1r). We also identified cell clusters corresponding to perivascular cells (pericyte, arterial smooth muscle cell, astrocyte), immune cells (T cell, B cell, border-associated macrophage, Ccr2+ monocyte/macrophage), as well as smaller clusters of other parenchymal cells (neuron, oligodendrocyte, oligodendrocyte precursor cell, ependymal cell, fibroblast). Additionally, we recovered a large population of choroid plexus cells showing high expression of Folr1, Enpp2, and Kcnj13. While interesting, this did not come as a surprise since the choroid plexus is highly vascularized with CD31+ endothelial cells [49, 50] and suggests that our technique did a good job isolating cells associated with the blood-cerebrospinal fluid barrier. Notably, the number of cells recovered per cluster were similar between HC and CSD samples (Additional file 1: Fig. S2c). A closer look the cell clusters recovered indicated that we recovered BVAC populations including EC populations, microglia, choroid plexus cells, pericytes, arterial smooth muscle cells, astrocytes, and border-associated macrophages (Fig. 2c), confirming that our isolation method effectively isolates and enriches for BVAC cells.

### Endothelial and microglia cell heterogeneity within BVACs

Brain EC and microglial dysfunction are implicated in many central nervous system pathologies. The brain contains diverse populations of ECs (i.e., capillary EC, arterial EC, venous EC) and microglia (i.e., homeostatic microglia, disease-associated microglia, activated microglia) that exhibit distinct transcriptional profiles. Yet, low EC and microglia yields following scRNAseq can lead to the grouping of diverse populations of ECs and microglia into single clusters, thus limiting the analyses that can be conducted on these cell populations. Of the 12,744 cells recovered from our BVAC isolation and sequencing, we obtained 4,208 ECs (33.0%) and 3,993 microglia (31.3%), indicating that our isolation technique successfully enriched for ECs and microglia. This allowed for finer unsupervised clustering of these cell populations into distinct cell populations that better represent the EC and microglial diversity in the brain (Additional file 1: Fig. S5).

**Fig. 3:**
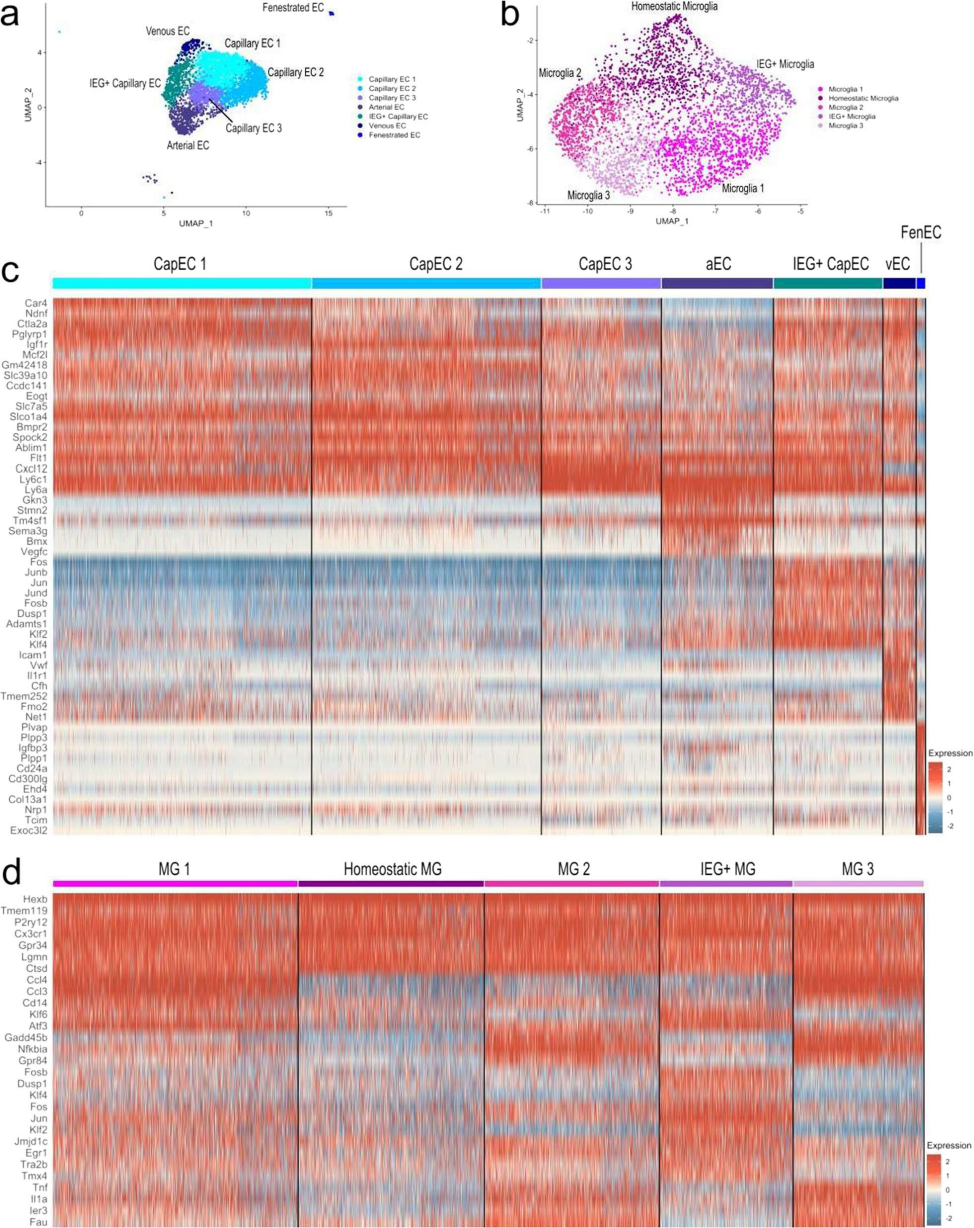
Deeper examination of endothelial and microglia gene expression reveals further cell type heterogeneity. **a** UMAP subset of 4208 cells depicting endothelial cell clusters. **b** UMAP subset of 3993 cells depicting microglia cell clusters. **c** Heatmap of endothelial cell signature gene expression compared to all other endothelial cell clusters. Gene expression patterns were used to identify 7 distinct endothelial cell clusters. **d** Heatmap of microglia signature gene expression compared to all other microglia clusters. Gene expression patterns were used to identify 5 distinct microglia clusters. For heatmaps, increased expression is colored red and decreased expression is colored blue. *CSD*  chronic social defeat; *HC*  home cage; *QC*  quality control; *UMAP* uniform manifold approximation and projection; *EC*  endothelial cell; *IEG* immediate-early gene; *SMC*  smooth muscle cell; *OPC*  oligodendrocyte precursor cell; *CapEC*  capillary endothelial cell; *aEC*  arterial endothelial cell; *vEC* venous endothelial cell; *FenEC* fenestrated endothelial cell; *MG* microglia

We isolated endothelial and microglia cells for further analysis. The 4208 ECs were further divided into 7 distinct clusters (Fig. 3a) based on their gene expression patterns (Fig. 3c). These clusters included distinct populations of arterial ECs (Gkn3, Stmn2, Bmx), venous ECs (Icam1, Vcam1, Vwf), and fenestrated ECs (Plvap, Plpp1, Cd24a). Capillary ECs made up the greatest proportion of recovered ECs, most likely due to their high abundance in the brain, and these were divided into three distinct capillary EC clusters based on their expression levels of Slc7a5, Slc16a1, Mfsd2a, Car4, and Cxcl12. A distinct cluster of capillary ECs expressed higher levels of immediate-early genes (IEGs) (i.e., Fos, Jun, Junb), and was thus termed IEG+ capillary EC. This limited population of ECs may be a result of our isolation technique, as enzymatic and mechanical isolation techniques may alter the transcriptional profiles of some cells [22, 51, 52]. The 3,993 microglia were further divided into 5 distinct clusters (Fig. 3b) based on their gene expression patterns (Fig. 3d). Homeostatic microglia expressed high levels of canonical homeostatic microglial genes including Cx3cr1, Hexb, Tmem119, P2ry12, Csf1r, C1qa, Tmem119, and Ctss. Similar to the clustering of the recovered ECs, a distinct population of microglia expressed higher levels of IEGs (i.e., Fos, Fosb, Erg1), and was thus termed IEG+ microglia. The remainder of the microglia expressed homeostatic microglial genes to a lower level compared to the homeostatic microglia and were divided into 3 distinct clusters based on their differing expression patterns of Ccl3, Ccl4, Il1a, Gadd45, Nfkbia, and Grp84. Overall, our isolation technique can isolate large populations of neurovascular endothelial and microglial cell populations that are rarely recovered by other single cell isolation techniques.

### CSD disrupts neurovascular integrity and activates immune pathways in BVACs

Mice susceptible to CSD show deficits in social behaviors that are known to coincide with anxiety-like and depressive-like behaviors measured in the open field, light/dark, sucrose preference, forced swim, and tail suspension tests [25]. Therefore, behavioral performance on the SI test was used to evaluate asocial, anxiety-like, and depressive-like behaviors following 14 days of CSD. Mice chosen for scRNAseq showed a significant reduction in social exploration in the SI test, as measured by the SI quotient (Unpaired t-test with Welch's correction, t = 5.22, p = 0.001, Fig. 1a). Heatmap tracings of mouse movement during the SI test indicated that mice subjected to CSD showed a reduced preference for the mouse-containing cylinder (Fig. 1b).

**Fig. 4:**
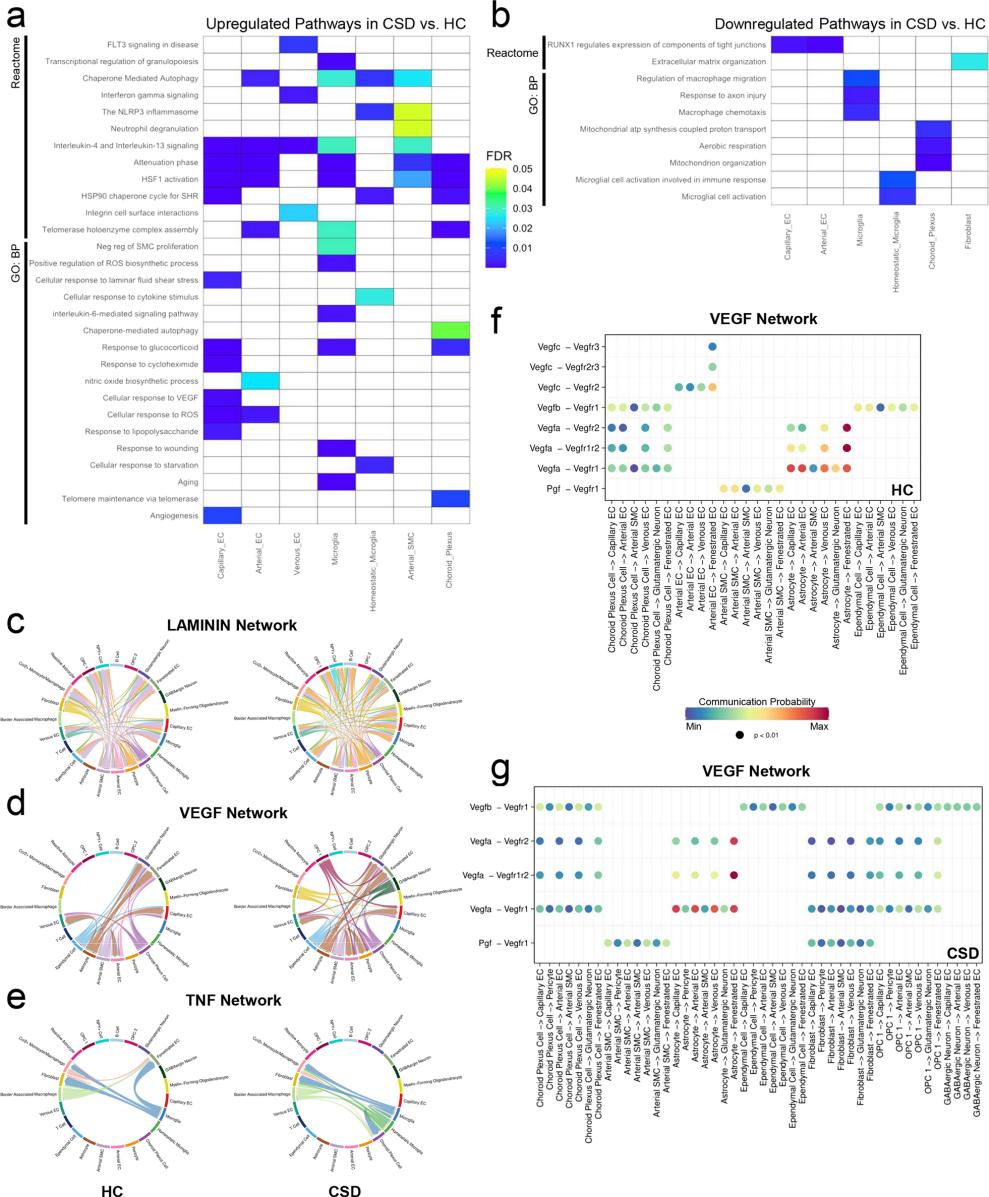
Pathway enrichment analysis reveals vascular dysfunction and immune system activation following CSD. **a**, **b** Analysis of differentially expressed (DE) genes (CSD vs. HC) in Reactome and Gene Ontology: Biological Processes (GO: BP) identity key upregulated (**a**) and downregulated (**b**) pathways. Only a subset of significant (FDR < 0.05) pathways is shown. **c**–**e** Chord diagrams showing LAMININ (**c**), VEGF (**d**), and TNF (**e**) cell–cell communication networks for HC and CSD inferred from CellChat. **f**, **g** Bubble plots comparing inferred ligand-receptor interactions within the VEGF network between HC (**f**) and CSD (**g**) for each cell type calculated from CellChat. *GO: BP*  Gene Ontology: Biological Processes; *FDR* false discovery rate; *SHR* steroid hormone receptor; *EC* endothelial cell; *SMC* smooth muscle cell; *OPC* oligodendrocyte precursor cell; *VEGF* vascular endothelial growth factor; *TNF* tumor necrosis factor; *HC* home cage; *CSD* chronic social defeat

We wanted to know how BVAC transcriptomes were altered due to CSD and how these changes may contribute to psychosocial stress-induced brain pathology. Therefore, we conducted differential gene expression analysis using the Seurat FindMarkers function (Additional file 3) and used these genes to probe for enriched pathways using Reactome and Gene Ontology: Biological Processes (GO: BP). For these analyses, Capillary EC clusters 1–3 were combined into one capillary EC cluster and microglia clusters 1–3 were combined into one Microglia cluster to maximize the number of cells in each cluster. A subset of upregulated Reactome and GO: BP pathways that reached significance (false discovery rate, FDR < 0.05) following CSD is shown in Fig. 4c, d, and a complete list of significant pathways can be found in Additional files 4 and 5.

Notably, CSD induced broad stress responses in multiple BVACs including capillary EC, arterial EC, microglia, homeostatic microglia, and choroid plexus cells as evidenced by an upregulation of pathways associated with cellular responses to wounding, fluid shear stress, glucocorticoids, starvation, aging, and lipopolysaccharides. Similarly, capillary and arterial EC populations showed increased responses to reactive oxygen species (ROS) and nitric oxide following CSD. CSD also led to an increase in pathways associated with autophagy and the heat shock protein response in many BVAC populations. Interleukin (IL) signaling pathways were also found to be upregulated following CSD, notably IL-4 and IL-13; these cytokines have diverse, context-dependent immunoregulatory roles in the brain and have been associated with both pro-inflammatory and anti-inflammatory responses [53–56]. Pathways involving vascular healing, like angiogenesis and response to vascular endothelial growth factor (VEGF), were also upregulated in the capillary EC, suggesting that the capillaries may be critical sites for vascular repair following CSD. While upregulation of damage response pathways seemed to predominate in the capillary and arterial EC populations, pathways involving interferon gamma signaling and integrin cell surface interactions were only elevated in the venous EC population following CSD, suggesting that veins may exhibit alterations in vascular permeability [57] and be a site of peripheral immune cell entry into the brain and perivascular spaces [58]. Interestingly, we also observed enrichment in pathways associated with neutrophil degranulation specifically in the arterial SMC population suggesting that neutrophils mobilized during psychosocial stress [42, 59] or the contents released by neutrophil degranulation may cross the BBB at microbleed sites and act on arterial SMCs (Fig. 4a). In capillary and arterial EC populations, CSD also led to the downregulation of pathways implicated in tight junction regulation, highlighting the brain-barrier breakdown caused by CSD [24]. Also downregulated in response to CSD were pathways involving fibroblast extracellular matrix organization as well as choroid plexus cell metabolism (Fig. 4b).

In microglia, CSD led to upregulation of pathways that contribute to microglial activation, including ROS biosynthesis [27, 60] and IL-6 signaling [61] (Fig. 4a). Microglia populations also showed reductions in pathways associated with activation and response to injury (Fig. 4b). These opposing changes in microglia may highlight the simultaneous states of damage and healing that likely exist during CSD. Overall, our results suggest that CSD impacts the transcriptional landscape of BVACs leading to alterations in pathways surrounding the cellular response to stress, brain-barrier dysfunction, vascular repair, and immune activation.

Since neurovascular cells are in close proximity, and likely communicating with each other at the blood–brain interface, we wanted to probe cell–cell communication networks in BVAC populations and determine if these interactions are altered by chronic psychosocial stress. Therefore, we computed cell–cell communication networks using CellChat [48], a curated database for inferring cell–cell communication and ligand-receptor interactions. For these analyses, the IEG+ Capillary EC, IEG+ Microglia, and Arterial SMC/Pericyte Doublet clusters were removed since the transcriptome of these cells may be influenced by our isolation technique or the presence of doublets. To validate the ability for CellChat to infer cell–cell communication networks in neurovascular cells, we began by computing interaction networks for all cells independent of stress experience. A total of 3,221 interactions contributing to 71 network pathways were inferred (Additional file 6). Notably, we found that EC populations uniquely contributed to the PECAM1 (CD31) network driven by Pecam1-Pecam1 interactions (Additional file 1: Fig. S6a–c) and that microglia were dominant recipients in the colony stimulating factor 1 (CSF1) network driven by Csf1-Csf1r and Il34-Csf1r interactions (Additional file 1: Fig. S6d–f). Taken together, this suggests that CellChat correctly predicts established EC and microglia cell–cell communication and ligand-receptor interactions in BVACs.

We then computed cell–cell interaction networks in HC and CSD BVAC populations and found that more interactions (HC: 2,644, CSD: 3,865) and network pathways (HC: 68, CSD: 78) were inferred in the stress condition, suggesting that a cell–cell communication networks may be altered by CSD (Additional file 6). Since previous analyses revealed that CSD alters pathways associated with brain-barrier dysregulation, vascular healing, and immune activation in BVAC populations (Fig. 4a, b), we explored cell–cell communication networks and ligand-receptor interactions that may contribute to these pathways. We found that the broad cell–cell interactions within the Laminin network, critical for neurovascular basement membrane and BBB integrity [62] and oligodendrocyte survival and myelination [63], VEGF network, and tumor necrosis factor (TNF) network were increased following CSD (Fig. 4c–e). Further exploration into EC tight junction-related networks, including the junctional adhesion molecule (JAM) and occludin (OCLN) networks, showed increased cell–cell interactions and a shift toward capillary EC involvement following CSD (Additional file 1 Fig. S7a–d). Given that CSD likely encompasses simultaneous states of both brain vascular dysfunction and healing, the increased tight junction-related interactions observed following CSD likely reflect the restoration of capillary brain-barrier integrity by EC repair processes following CSD-induced vascular damage. Deeper investigation into the predicted cellular contributors and ligand-receptor interactions within the VEGF network revealed that EC populations are the main recipient of VEGF signaling and that CSD lead to an increase in the number of BVAC populations sending VEGF signals to EC clusters (Fig. 4f, g), further supporting the enrichment in vascular repair pathways uncovered by pathway analysis (Fig. 4a). Interestingly, some inferred cell–cell communication networks were unique to CSD, including the CX3C network (Additional file 1: Fig. S7e, f), suggesting that Cx3cl1-Cx3cr1 fractalkine signaling may play an important role in microglial responses to CSD. Together, these inferred interactions suggest that CSD disrupts cell–cell communication networks within BVAC populations that contribute to stress-induced neurovascular pathology and repair.

## Discussion

Deep examination of the cell types that comprise the brain vasculature in health and disease has proven challenging due to inadequate methods to isolate the cells for downstream transcriptional analysis. Here, we developed a technique to isolate and enrich for BVACs and applied the technique to interrogate the transcriptional profiles of 12,744 cells from fresh male mouse brain tissue following CSD and non-stress conditions. We identified multiple distinct cell populations representing ECs and microglia, as well as less commonly recovered cell populations including mural and immune cells. Our high recovery of ECs and microglia enabled us to further subclassify these cell populations in a manner that better represents their diversity within the brain.

Pathway analysis of differentially expressed genes in male mice following 14 days of CSD revealed that BVAC populations adopt unique transcriptional profiles in response to CSD. Most notably, our work revealed that chronic psychosocial stress triggers stress-response mechanisms in many different BVAC populations. In line with recent reports showing that chronic psychosocial stress induces the formation of cerebrovascular microbleeds [26] and elevates microglial ROS activity [27], CSD led to the downregulation of RUNX1-mediated tight junction regulation and upregulation of pathways associated with microglial activation, including ROS biosynthesis [27, 60] and IL-6 signaling [61]. This is supported by evidence suggesting that Laminin, JAM, OCLN, and TNF cell–cell communication networks are dysregulated following CSD. Thus, we suggest that a critical microglia-EC axis exists that may contribute to vascular dysfunction following chronic psychosocial stress, especially since recently characterized microglial populations closely interact with the brain vasculature [64, 65]. While broad immune signaling has been implicated in multiple neurological pathologies characterized by neurovascular dysfunction [66], our work highlights IL-4 and IL-13 signaling as possible key immune pathways. Interestingly, IL-4 and IL-13 have distinct context-dependent immunoregulatory roles in the brain and have been associated with both pro-inflammatory and anti-inflammatory responses [53–56], suggesting that they may be capable of mediating both chronic psychosocial stress-induced neurovascular damage and healing. Additionally, our work highlights an enrichment in venous EC-specific interferon gamma signaling, further supporting that these cells exhibit alterations in vascular permeability [57] in response to CSD and may serve as a site of peripheral immune cell entry into the brain and perivascular spaces [58]. Our work further reveals that the cellular mechanisms activated in response to chronic psychosocial stress are dynamic. Angiogenic processes, possibly driven via VEGF signaling [67], coupled with reduced microglial activation patterns were found alongside pro-injury response signals, suggesting that patterns of damage and healing can occur simultaneously at the brain vasculature. To further expand upon the dynamic nature of neurovascular cell responses to CSD, future studies should isolate and examine neurovascular cells at multiple time points throughout the chronic psychosocial stress period as this would provide interesting insight into the temporal nature of transcriptional changes induced by CSD. Additionally, the use of fluorescence in-situ hybridization will be necessary to further validate the pathways uncovered by our sequencing analysis. To better address sex as a biological variable, novel methods have been developed that allows for CSD in female mice [68, 69]. Therefore, the inclusion of female mice in future CSD studies should be explored to determine if sex plays a role in the transcriptional changes observed following CSD in neurovascular cells, especially since sex has recently been shown to influence BBB integrity [70] and the transcriptional landscape associated with chronic stress and major depression [71]. Overall, these results suggest that BVACs undergo unique transcriptional changes in response to CSD and are likely to participate in the progression of psychosocial-stress mediated pathologies. Further, these findings provide further support for therapeutic approaches targeting the cerebrovasculature in neuropathology.

Neurovascular dysfunction has been observed in numerous human disease cases [9, 15, 16, 72, 73]. Therefore, a deeper understanding of the transcriptional patterns that govern the cerebrovasculature in homeostasis and disease will be critical to understanding how its dysfunction contributes to pathology. While recent studies are beginning to probe the human brain vasculature in neurodegenerative diseases [74, 75], the current landscape of human and mouse transcriptional studies in psychiatric and affective disorders, like MDD, focus mostly on neuronal and synaptic structure [76, 77] and thus fall short in addressing the vascular components of these pathologies. Our technique adds to a growing body of methods that aim to interrogate the mouse [17, 18, 46, 78–83] and human [74, 75, 84] cerebrovascular system in health and disease. Current methods using fluorescence-activated cell sorting have been employed to effectively isolate neurovascular cells with high specificity, sample throughput, and cellular recovery [22, 46, 82, 83]. Additionally, the emergence of recently developed laser capture microdissection methods allow for highly specific neurovascular cell capture at a snapshot in time without long incubation times [20, 85, 86], but at the expense of laborious dissection time and low sample throughput [22]. In comparison to recently reported methods, our technique for live cell isolation carries notable benefits in that it is fast, successfully enriches for many populations of vascular-associated cells, generates a pool of single cells that are highly viable and pure, and does well to dissociate closely connected cells into single cells, thus reducing the issue of doublet contamination. Despite these benefits, we acknowledge that our method is not exempt from common pitfalls inherent to single-cell isolation procedures. The use of antibody-coupled magnetic beads for positive neurovascular selection relies on efficient antibody-epitope binding, a strategy also relied upon by other single cell isolation methods, including fluorescence-activated cell sorting. Unfortunately, this interaction that can be disrupted by enzymatic and mechanical digestion steps used in many isolation procedures—thus, possibly reducing overall cellular yields despite achieving high purity [22]. Similarly, the use of enzymatic digestion may unintentionally alter the transcriptional profile of cells prior to sequencing [52] which has encouraged some recent studies to employ gradient or density filtration methods as additional approaches [87]. Therefore, the use of digestion or filtration steps must be carefully considered, especially when utilizing antibody-based isolation approaches. Overcoming these barriers during single cell isolation will be critical to increasing cell recovery and preserving cell integrity of neurovascular cells in future studies. Additionally, the employment of recently developed sequencing strategies, like spatial transcriptomics, will uniquely allow for selective targeting and interrogation of neurovascular regions undergoing CSD-induced damage and repair processes. Overall, the use of multiple isolation techniques and sequencing technologies will be required to gain a comprehensive understanding of the neurovascular landscape in health and disease.

## Conclusions

In summary, our work provides a technique to efficiently isolate and enrich for cell populations comprising the cerebrovasculature that can be translated to other models of pathology. Using this technique paired with scRNAseq, we showed that CSD alters the transcriptional profile of multiple BVAC populations and sheds light on mechanisms that may link psychosocial stress to neurovascular pathology and healing. Overall, our findings suggest that neurovascular-associated cells are active players in psychosocial stress-induced neuropathology and healing. Thus, further efforts should focus on investigating the roles of neurovascular-associated cells to determine if novel therapeutic targets exist to treat stress-induced neuropathology.

## Supplementary Information

**Additional file 1: Figure S1.** Pre-study social interactionphenotyping identifies animals with abnormal baseline social behavior that were removed from the study.The SI quotient for all animals. The SI quotient was calculated by dividing the time spent investigating the cylinder containing the CD-1 mouse by the time spent investigating the empty cylinder.The total non-interaction time for all animals. The total non-interaction time was calculated by adding the total time spent in each of the four area corners. The circled-times operator symbolindicates 6 animals that were removed from the study due to abnormal behavior. The filled circle symbol indicates animalsthat were used for the remainder of the study. Abbreviations: SI = social interaction. **Figure S2.** Similar cell type populations are detected across HC and CSD samples following QC and integration.UMAPs following QC and integration, split by condition.UMAPs following QC and integration split by sample.Percentage of cells for each identified cell type cluster to the total number of cells acquired split by condition. Error bars = standard error of the mean. Abbreviations: CSD = chronic social defeat; HC home cage; QC = quality control; UMAP = uniform manifold approximation and projection; SEM = standard error of the mean; EC = endothelial cell; IEG = intermediate early gene; SMC = smooth muscle cell; OPC = oligodendrocyte precursor cell. **Figure S3.** Signature gene expression identifies and classifies BVAC cell clusters. Heatmap of signature gene expression compared to all other clusters used to identify 26 cell type clusters recovered from the neurovascular isolation. Increased expression is colored red and decreased expression is colored blue. Colorbar corresponds with identified cell type clusters. Abbreviations: EC = endothelial cell; IEG = intermediate early gene; SMC = smooth muscle cell; OPC = oligodendrocyte precursor cell. **Figure S4.** Key signature genes identify and classify BVAC clusters. Feature plots depicting gene expression level of key signature genes for neurovascular cell clusters. Abbreviations: EC = endothelial cell; Cap EC = capillary endothelial cell; aEC = arterial endothelial cell; vEC = venous endothelial cell; FenEC = fenestrated endothelial cell; MG = microglia; IEG = intermediate early gene; ChPlex = Choroid Plexus; aSMC = arterial smooth muscle cell; AC = astrocyte; Epend = ependymal; BAM = border associated macrophage; Mo/M0 = monocyte/macrophage; rAC = reactive astrocyte; OPC = oligodendrocyte precursor cell; Glu+ Neu = glutamatergic neuron; GABA+ Neu = GABAergic neuron; mOG = myelin-forming oligodendrocytes. **Figure S5.** Unsupervised sub-clustering of EC and Microglia clusters. UMAP of 12,744 cells following QC and integration from all CSD and HC samples showing sub-clustering of capillary endothelial cell and microglia clusters. **Figure S6.** CellChat predicts well-known endothelial cell and microglia cell–cell communication and ligand-receptor interactions in neurovascular cells.PECAM1 network for all cellsvisualized with Cell–cell communication chord diagram, centrality heatmap, and contributing ligand-receptor pairspredicted by CellChat.CSF1 network for all cell typesvisualized with Cell–cell communication chord diagram, centrality heatmap, and contributing ligand-receptor pairspredicted by CellChat. Abbreviations: CSF1 = colony stimulating factor 1; EC = endothelial cell; SMC = smooth muscle cell; OPC = oligodendrocyte precursor cell; HC = home cage; CSD = chronic social defeat. **Figure S7.** Predicted cell–cell communication and ligand-receptor interactions implicate BBB dysfunction and immune activation following CSD.Chord diagrams showing JAMand OCLNcell–cell communication networks for HC and CSD inferred from CellChat.Centrality heatmaps for JAMand OCLNnetworks comparing predicted cellular contribution between HC and CSD for each cell type calculated from CellChat.Chord diagram showing the CSD-specific CX3C cell–cell communication network inferred from CellChat.Ligand-receptor pairs contributing to the CSD-specific CX3C network predicted by CellChat. JAM = junctional adhesion molecule; OCLN = occludin; EC = endothelial cell; SMC = smooth muscle cell; OPC = oligodendrocyte precursor cell; HC = home cage; CSD = chronic social defeat

**Additional file 2: Table S1.** Identifying key signature genes for BVAC clusters.

**Additional file 3:** **Table S2.** Containing a full list of differentially expressed genes between across all cell clusters.

**Additional file 4: Table S3.** Containing enriched pathways from Reactome for all clusters.

**Additional file 5: Table S4.** Containing enriched pathways from Gene Ontology: Biological Processes for all clusters

**Additional file 6: Table S5.** containing CellChat network predictions for combined, HC, and CSD

## Data Availability

Raw sequencing data associated with this study have been deposited in the NCBI Gene Expression Omnibus (GEO) under accession code GSE210577. The scRNAseq R workflow and code used in this study are available at GitHub (https://github.com/josh-samuels1200/Samuels-et-al_BVAC_scRNAseq_Analysis) and upon reasonable request from the corresponding author.

## Abbreviations

AC: Astrocyte
aEC: Arterial endothelial cell
BAM: Border-associated macrophage
BBB: Blood–brain barrier
BSA: Bovine serum albumin
BVAC: Brain vascular-associated cell
CapEC: Capillary EC
ChPlex: Choroid plexus
CSD: Chronic social defeat
CSF: Cerebrospinal fluid
CSF1: Colony stimulating factor 1
DMEM: Dulbecco's modified eagle medium
EC: Endothelial cell
EDTA: Etheylenediaminetetraacetic acid
FDR: False discovery rate
FenEC: Fenestrated endothelial cell
GO: BP: Gene ontology: biological processes
HBSS: Hank’s balanced salt solution
HC: Home cage
IEG: Immediate-early genes
IL: Interleukin
JAM: Junctional adhesion molecule
M: Mouse
MDD: Major depressive disorder
MG: Microglia
O: Object
OCLN: Occludin
OPC: Oligodendrocyte precursor cell
PBS: Phosphate buffered saline
QC: Quality control
ROS: Reactive oxygen species
TNF: Tumor necrosis factor
scRNAseq: Single-cell RNA sequencing
SHR: Steroid hormone receptor
SI: Social interaction
SMC: Smooth muscle cell
UMAP: Uniform manifold approximation and projection
vEC: Venous endothelial cell
VEGF: Vascular endothelial growth factor
