# Supplementary material for: Chronic social defeat alters brain vascular-associated cell gene expression patterns leading to vascular dysfunction and immune system activation
Source: J Neuroinflammation. 2023 Jun 28;20:154. doi: 10.1186/s12974-023-02827-5 (PMC10303797; doi:10.1186/s12974-023-02827-5)
Supplement: Supplementary file 1 — Additional file 1: Figure S1. Pre-study social interactionphenotyping identifies animals with abnormal baseline social behavior that were removed from the study.The SI quotient for all animals. The SI quotient was calculated by dividing the time spent investigating the cylinder containing the CD-1 mouse by the time spent investigating the empty cylinder.The total non-interaction time for all animals. The total non-interaction time was calculated by adding the total time spent in each of the four area corners. The circled-times operator symbolindicates 6 animals that were removed from the study due to abnormal behavior. The filled circle symbol indicates animalsthat were used for the remainder of the study. Abbreviations: SI = social interaction. Figure S2. Similar cell type populations are detected across HC and CSD samples following QC and integration.UMAPs following QC and integration, split by condition.UMAPs following QC and integration split by sample.Percentage of cells for each identified cell type cluster to the total number of cells acquired split by condition. Error bars = standard error of the mean. Abbreviations: CSD = chronic social defeat; HC home cage; QC = quality control; UMAP = uniform manifold approximation and projection; SEM = standard error of the mean; EC = endothelial cell; IEG = intermediate early gene; SMC = smooth muscle cell; OPC = oligodendrocyte precursor cell. Figure S3. Signature gene expression identifies and classifies BVAC cell clusters. Heatmap of signature gene expression compared to all other clusters used to identify 26 cell type clusters recovered from the neurovascular isolation. Increased expression is colored red and decreased expression is colored blue. Colorbar corresponds with identified cell type clusters. Abbreviations: EC = endothelial cell; IEG = intermediate early gene; SMC = smooth muscle cell; OPC = oligodendrocyte precursor cell. Figure S4. Key signature genes identify and classify BVAC clusters. Feature plots de [file 12974_2023_2827_MOESM1_ESM.docx]

Chronic social defeat alters brain vascular-associated cell gene expression patterns leading to vascular dysfunction and immune system activation

Joshua D. Samuels^1*^, Madison L. Lotstein^1^, Michael L. Lehmann^1^, Abdel G. Elkahloun^2^, Subhadra Banerjee^3^, Miles Herkenham^1^

**Additional File 1: Supplemental figures.**

**
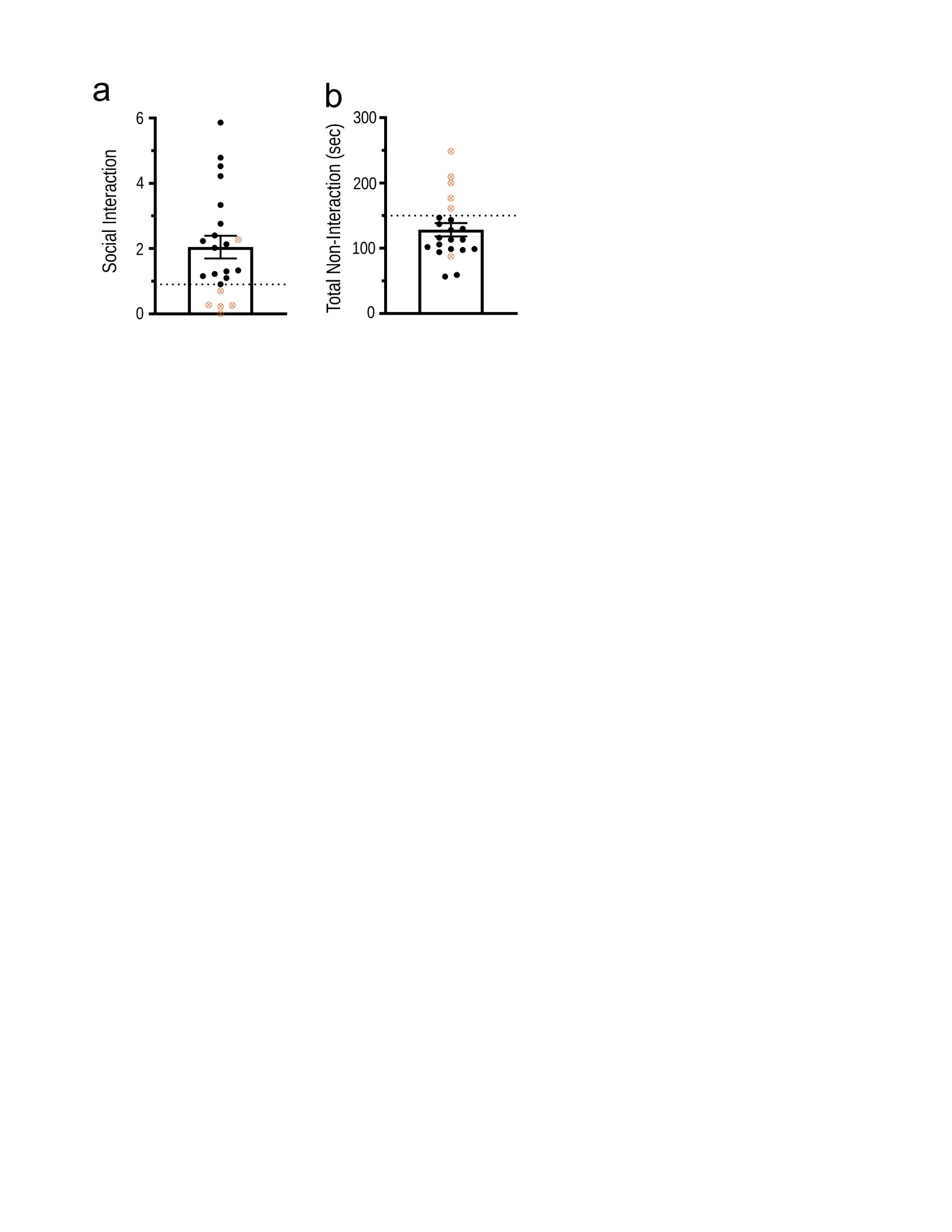
**

**Fig. S1 Pre-study social interaction (SI) phenotyping identifies animals with abnormal baseline social behavior that were removed from the study.** (a) The SI quotient for all animals (n = 22). The SI quotient was calculated by dividing the time spent investigating the cylinder containing the CD-1 mouse by the time spent investigating the empty cylinder. (b) The total non-interaction time for all animals. The total non-interaction time was calculated by adding the total time spent in each of the four area corners. The circled-times operator symbol (⛒) indicates 6 animals that were removed from the study due to abnormal behavior. The filled circle symbol indicates animals (n = 16) that were used for the remainder of the study. Abbreviations: SI = social interaction.

**
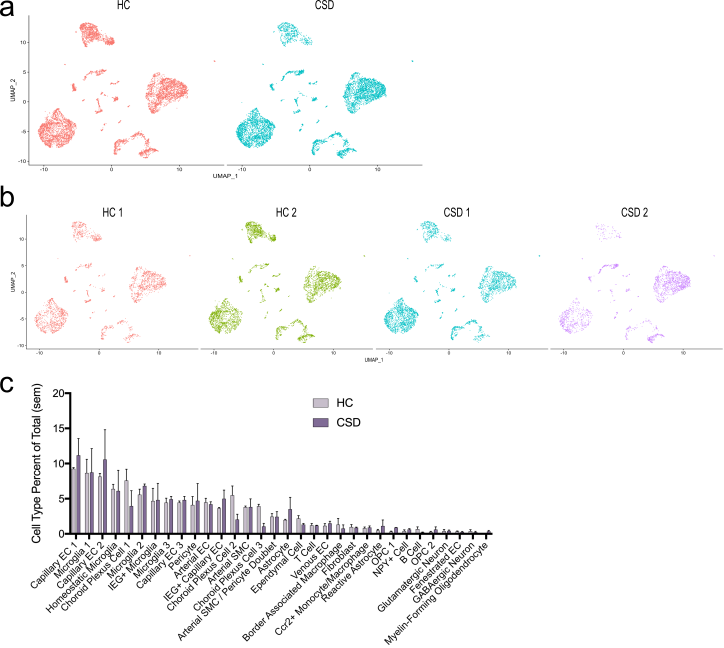
**

**Fig. S2 Similar cell type populations are detected across HC and CSD samples following QC and integration.** (a) UMAPs following QC and integration, split by condition (HC: 6,749 cells; CSD: 5,995 cells). (b) UMAPs following QC and integration split by sample (HC1: 2,547 cells; HC2: 4,202 cells; CSD1: 3,761 cells; CSD2: 2,434 cells). (c) Percentage of cells for each identified cell type cluster to the total number of cells acquired split by condition. Error bars = standard error of the mean. Abbreviations: CSD = chronic social defeat; HC home cage; QC = quality control; UMAP = uniform manifold approximation and projection; SEM = standard error of the mean; EC = endothelial cell; IEG = intermediate early gene; SMC = smooth muscle cell; OPC = oligodendrocyte precursor cell.


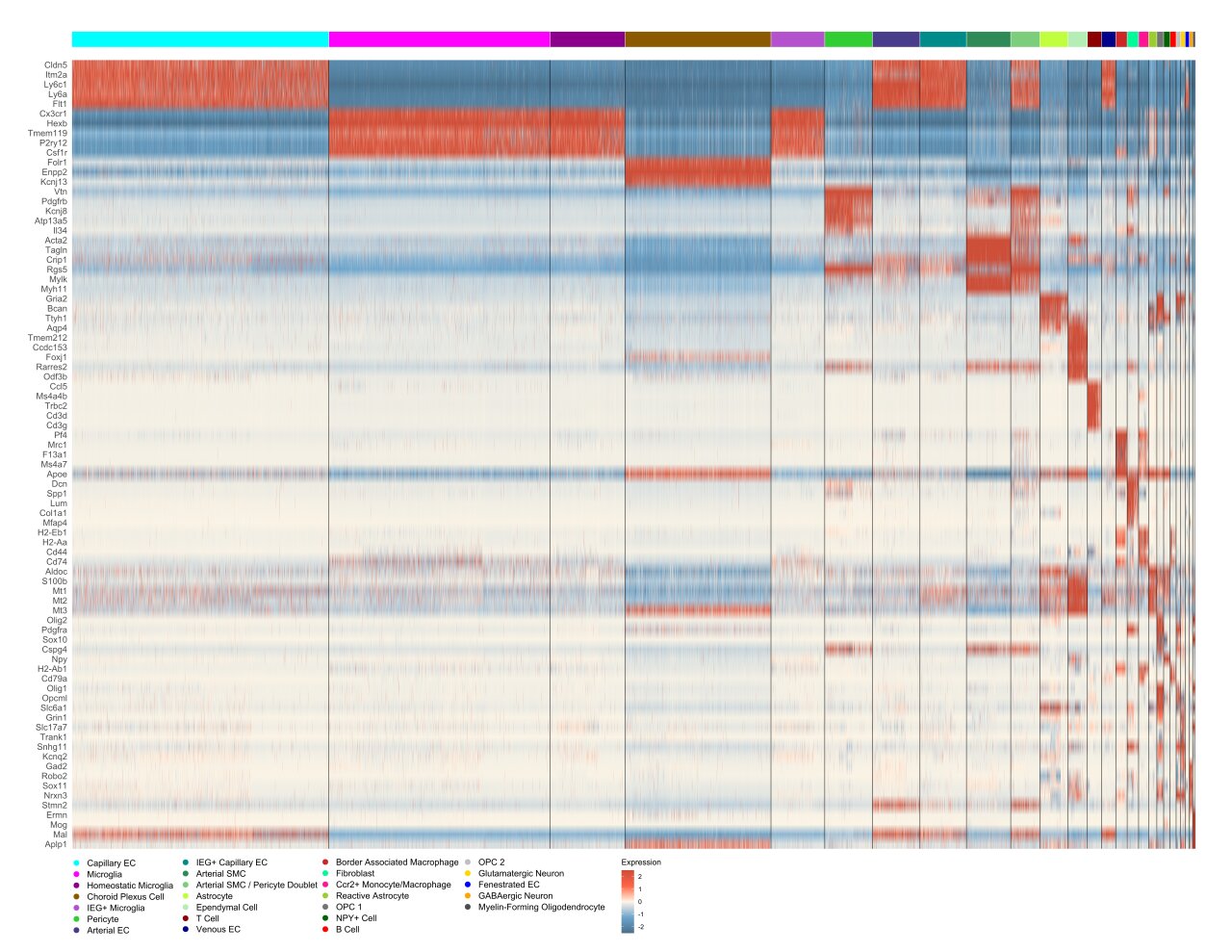


**Fig. S3 Signature gene expression identifies and classifies BVAC cell clusters.** Heatmap of signature gene expression compared to all other clusters used to identify 26 cell type clusters recovered from the neurovascular isolation. Increased expression is colored red and decreased expression is colored blue. Colorbar corresponds with identified cell type clusters. Abbreviations: EC = endothelial cell; IEG = intermediate early gene; SMC = smooth muscle cell; OPC = oligodendrocyte precursor cell.

**
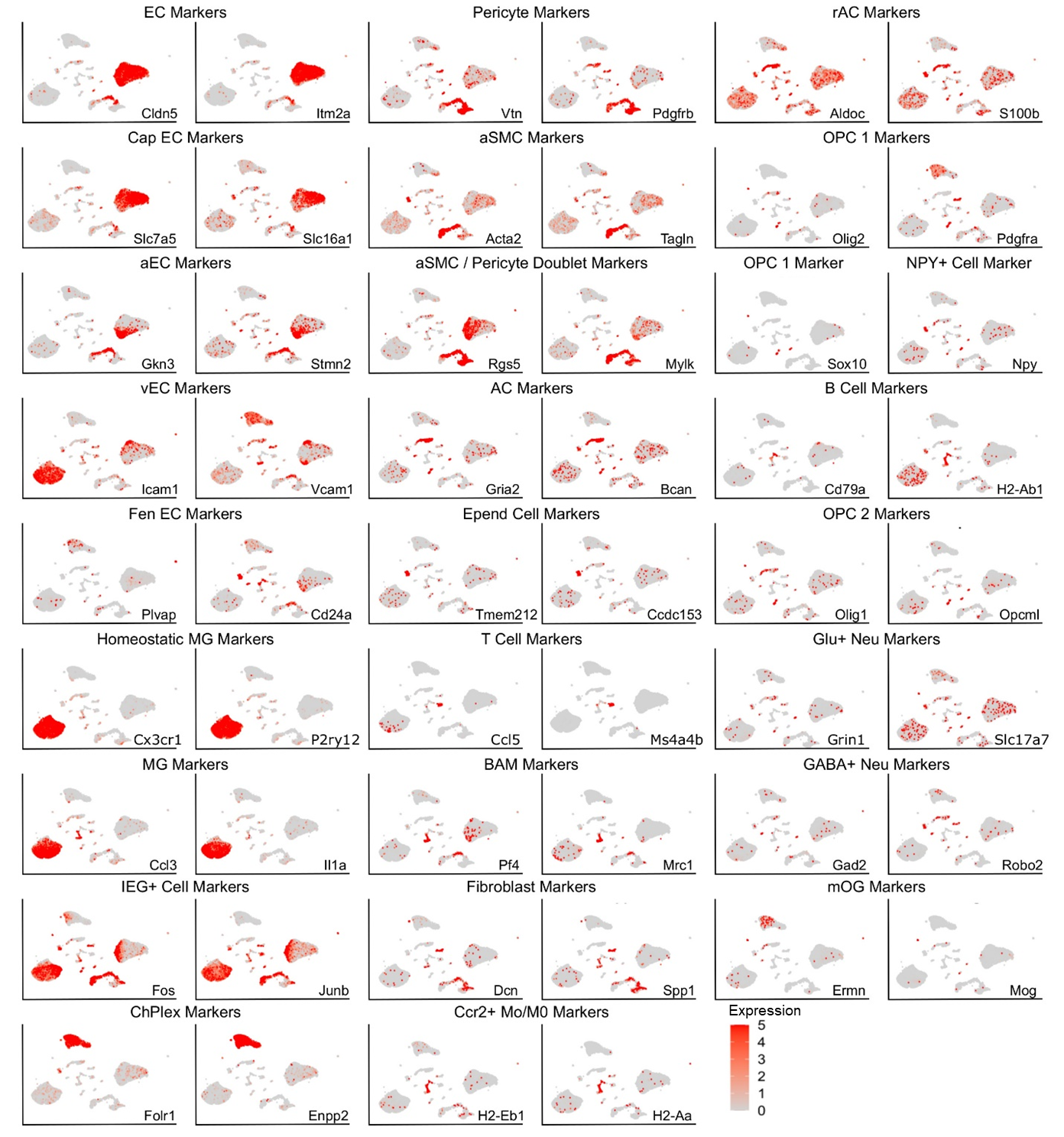
**

**Fig. S4 Key signature genes identify and classify BVAC clusters.** Feature plots depicting gene expression level of key signature genes for neurovascular cell clusters. Abbreviations: EC = endothelial cell; Cap EC = capillary endothelial cell; aEC = arterial endothelial cell; vEC = venous endothelial cell; FenEC = fenestrated endothelial cell; MG = microglia; IEG = intermediate early gene; ChPlex = Choroid Plexus; aSMC = arterial smooth muscle cell; AC = astrocyte; Epend = ependymal; BAM = border associated macrophage; Mo/M0 = monocyte/macrophage; rAC = reactive astrocyte; OPC = oligodendrocyte precursor cell; Glu+ Neu = glutamatergic neuron; GABA+ Neu = GABAergic neuron; mOG = myelin-forming oligodendrocytes.


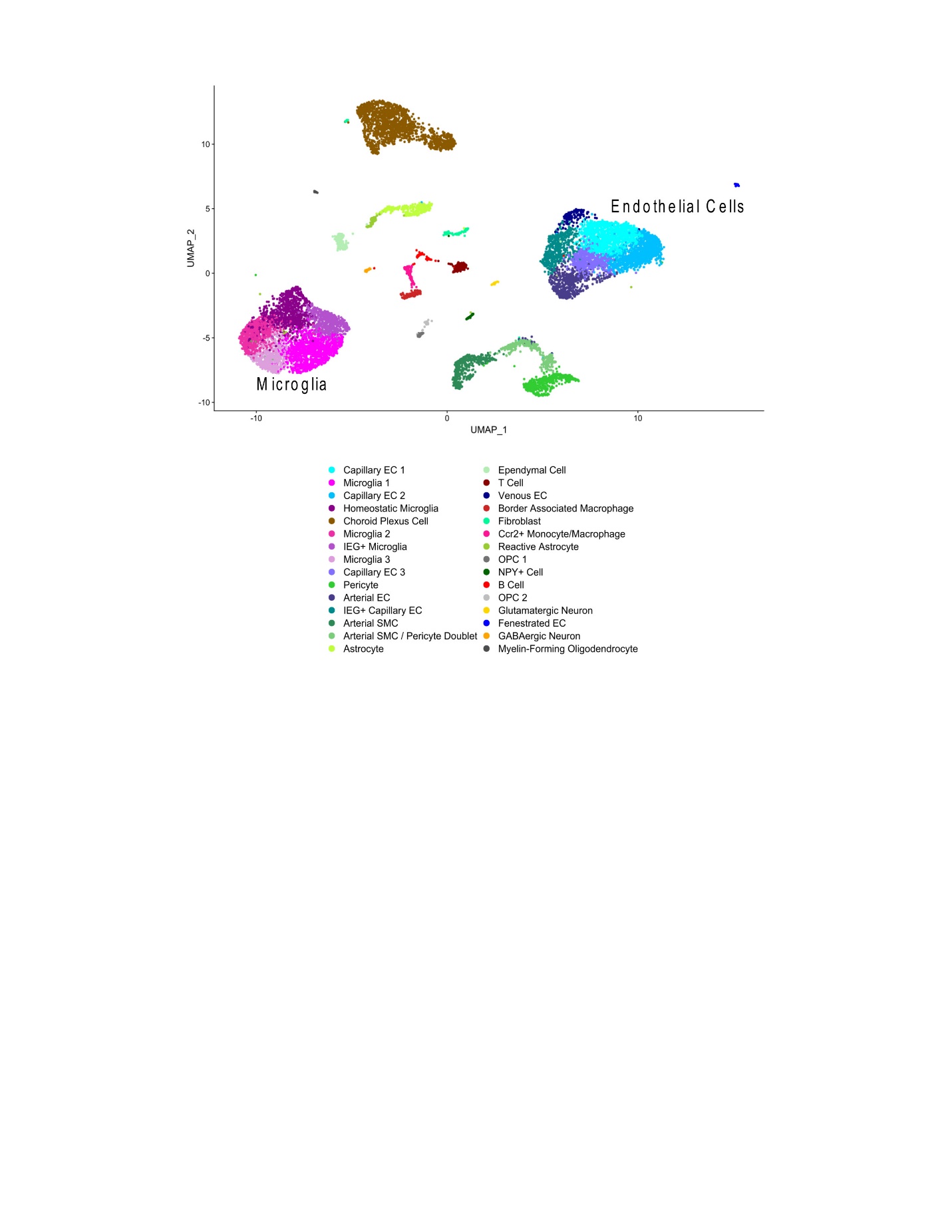


**Fig. S5 Unsupervised sub-clustering of EC and Microglia clusters.** UMAP of 12,744 cells following QC and integration from all CSD and HC samples showing sub-clustering of capillary endothelial cell and microglia clusters.


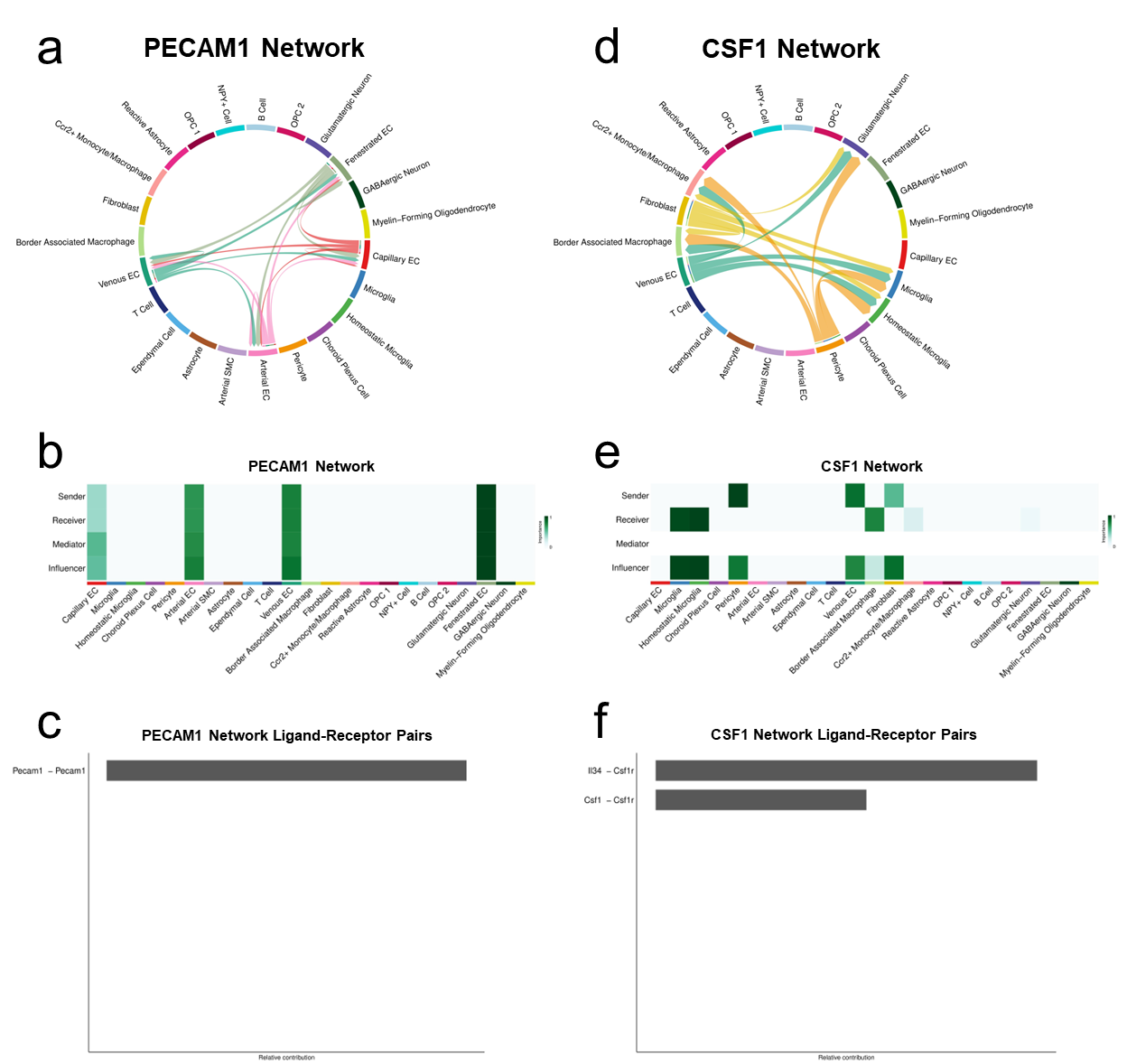


**Fig. S6 CellChat predicts well-known endothelial cell and microglia cell-cell communication and ligand-receptor interactions in neurovascular cells.** (a, b, c) PECAM1 network for all cells (HC and CSD combined) visualized with Cell-cell communication chord diagram (a), centrality heatmap (b), and contributing ligand-receptor pairs (c) predicted by CellChat. (d, e, f) CSF1 network for all cell types (HC and CSD combined) visualized with Cell-cell communication chord diagram (d), centrality heatmap (e), and contributing ligand-receptor pairs (f) predicted by CellChat. Abbreviations: CSF1 = colony stimulating factor 1; EC = endothelial cell; SMC = smooth muscle cell; OPC = oligodendrocyte precursor cell; HC = home cage; CSD = chronic social defeat.


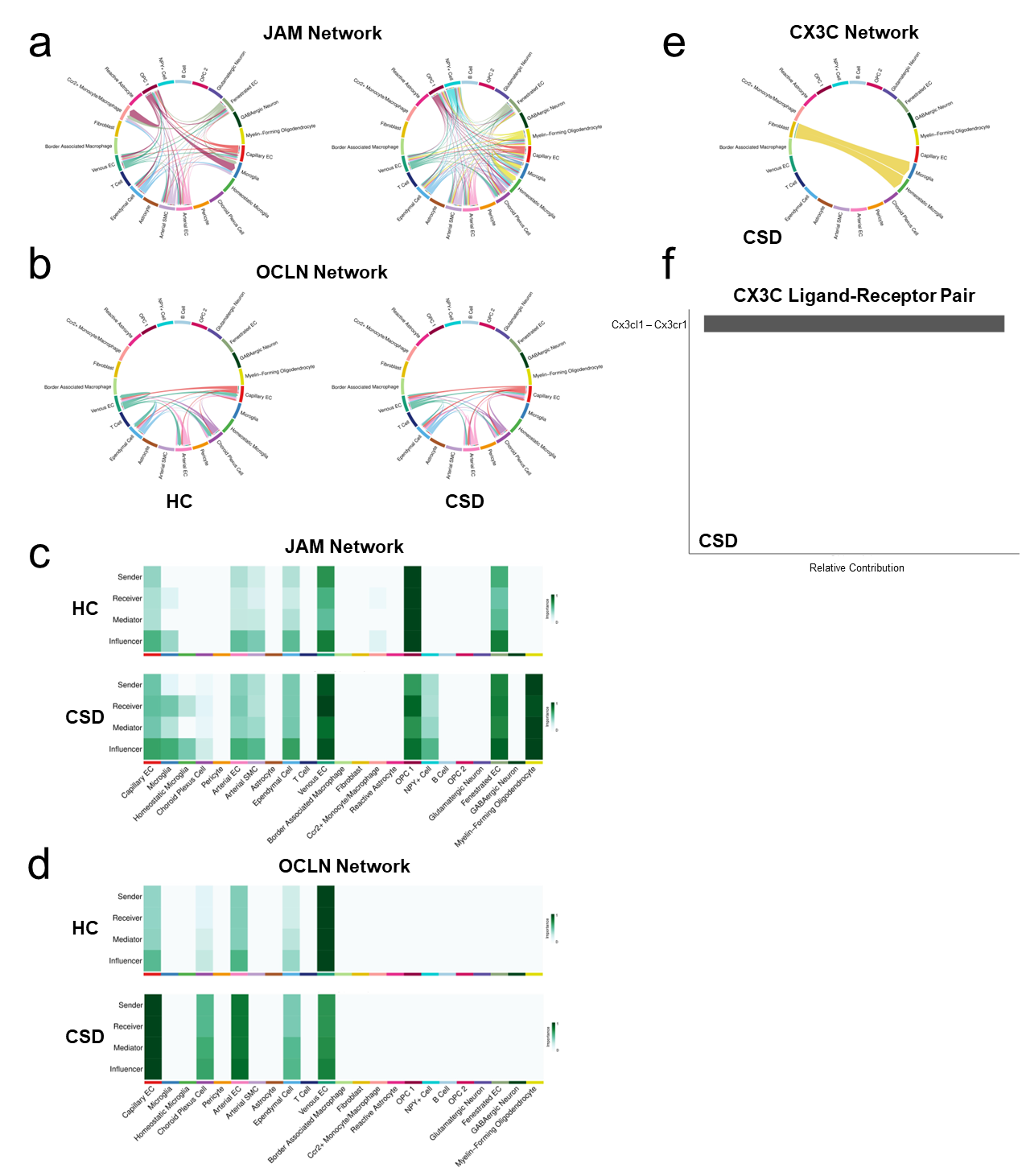


**Fig. S7 Predicted cell-cell communication and ligand-receptor interactions implicate BBB dysfunction and immune activation following CSD.** (a, b) Chord diagrams showing JAM (a) and OCLN (c) cell-cell communication networks for HC and CSD inferred from CellChat. (c, d) Centrality heatmaps for JAM (c) and OCLN (d) networks comparing predicted cellular contribution between HC and CSD for each cell type calculated from CellChat. (e) Chord diagram showing the CSD-specific CX3C cell-cell communication network inferred from CellChat. (f) Ligand-receptor pairs contributing to the CSD-specific CX3C network predicted by CellChat. Abbreviations: JAM = junctional adhesion molecule; OCLN = occludin; EC = endothelial cell; SMC = smooth muscle cell; OPC = oligodendrocyte precursor cell; HC = home cage; CSD = chronic social defeat.
