# Supplementary material for: Chronic social defeat alters brain vascular-associated cell gene expression patterns leading to vascular dysfunction and immune system activation
Source: J Neuroinflammation. 2023 Jun 28;20:154. doi: 10.1186/s12974-023-02827-5 (PMC10303797; doi:10.1186/s12974-023-02827-5)
Supplement: Supplementary file 2 — Additional file 2: Table S1. Identifying key signature genes for BVAC clusters. [file 12974_2023_2827_MOESM2_ESM.docx]

Chronic social defeat alters brain vascular-associated cell gene expression patterns leading to vascular dysfunction and immune system activation

Joshua D. Samuels^1*^, Madison L. Lotstein^1^, Michael L. Lehmann^1^, Abdel G. Elkahloun^2^, Subhadra Banerjee^3^, Miles Herkenham^1^

**Additional File 2: Table 1.**

| **Table S1. Key signature genes for BVAC cell clusters.** | | | |
| --- | --- | --- | --- |
|  | **Cell Type** | **Signature Genes** | |
| **Endothelial Cells** | Capillary EC 1 | *Cldn5, Itm2a, Ly6c1, Ly6a, Flt1* | *Slc7a5, Slc16a1, Mfsd2a* |
|  | Capillary EC 2 |  | *Slc7a5, Slc16a1, Mfsd2a* |
|  | Capillary EC 3 |  | *Slc7a5, Slc16a1, Mfsd2a* |
|  | Arterial EC |  | *Gkn3, Stmn2, Bmx* |
|  | Venous EC |  | *Icam1, Vcam1, Vwf* |
|  | Fenestrated EC |  | *Plvap, Plpp1, Cd24a, Esm1* |
|  | IEG+ Capillary EC |  | *Fos, Jun, Junb* |
| **Microglia** | Homeostatic Microglia | *Cx3cr1, Hexb, Tmem119, P2ry12, Csf1r* | *C1qa, Tmem119, Ctss* |
|  | Microglia 1 |  | *Ccl3, Ccl4* |
|  | Microglia 2 |  | *Ila, Gadd45, Nfkbia, Gpr84* |
|  | Microglia 3 |  | *Ccl3, Ccl4, Ila, Gadd45, Nfkbia, Gpr84* |
|  | IEG+ Microglia |  | *Fos, Fosb, Egr1* |
| **Perivascular Cells** | Pericyte | *Vtn, Pdgfrb, Kcnj8, Atp13a5* | |
|  | Arterial SMC | *Vtn, Pdgfrb, Acta2, Tagln, Tmp2, Mylk, Myh11* | |
|  | Arterial SMC/Pericyte Doublet | *Vtn, Pdgfrb, Mylk, Myh11* | |
|  | Astrocyte | *Gria2, Bcan, Ttyh1, Ntsr2* | |
|  | Reactive Astrocyte | *Aldoc, S100b, Mt1, Mt2, Mt3* | |
| **Immune Cells** | T Cell | *Ccl5, Ms4a4b, Cd3d, Trbc2* | |
|  | B Cell | *Cd74, Ly6d, H2-Ab1, Cd79a* | |
|  | Border Associated Macrophage | *Pf4, Mrc1, F13a1, Ms4a7* | |
|  | Ccr2+ Monocyte/Macrophage | *Ccr2, H2-Eb1, Cd44, H2-Aa* | |
| **Other Parenchymal Cells** | Ependymal Cell | *Tmem212, Ccdc153, Foxj1, Rarres2* | |
|  | Fibroblast | *Dcn, Spp1, Lum, Col1a1* | |
|  | Myelin-Forming Oligodendrocyte | *Ermn, Mog, Mal, Aplp1* | |
|  | OPC 1 | *Pdgfra, Olig2, Sox10* | |
|  | OPC 2 | *Pdgfra, Olig1, Opcml, Slc6a1* | |
|  | Glutamatergic Neuron | *Grin1, Slc17a7, Nrxn3, Trank1, Kcnq2* | |
|  | GABAergic Neuron | *Gad2, Robo2, Sox11, Nrxn3, Stmn2* | |
|  | Npy+ Cell | *Npy, Fabp7* | |
|  | Choroid Plexus Cell | *Folr1, Enpp2, Kcnj13, Ttr* | |
| *Abbreviations: EC = endothelial cell; IEG = intermediate early gene; SMC = smooth muscle cell; OPC = oligodendrocyte precursor cell.* | | | |
